# Water Kinetics and Performance of Concrete Internally Cured Using Waste Soda Residue

**DOI:** 10.3390/ma19194212

**Published:** 2026-10-02

**Authors:** Huaiyu Li, Kaiyue Zhao, Yao Wang, Yijun Zhou, Yunlong Zhou

**Affiliations:** College of Civil and Architectural Engineering, North China University of Science and Technology, Tangshan 063210, China

**Keywords:** soda residue, internal curing, concrete, absorption–desorption, macro performance

## Abstract

To mitigate early-age shrinkage cracking and enhance the durability of concrete, this study explores the valorization of soda residue, an industrial byproduct, as an internal curing agent. A novel soda residue-based internal curing agent material was synthesized via water washing and fly ash-assisted grinding. Concrete mixtures with varying soda residue contents were evaluated, revealing that the incorporation of 10% soda residue yielded optimal performance, characterized by significantly reduced autogenous shrinkage, superior frost resistance, and enhanced later-age compressive strength. The transition from macro performance to underlying mechanisms was elucidated through low-field nuclear magnetic resonance and calorimetry. The results demonstrate that the performance of soda residue-modified concrete is governed by a competition between the beneficial internal curing effect and the introduction of weak interfaces. At the optimal 10% dosage, the positive internal curing effect predominates. Specifically, the water kinetics—as evidenced by a distinct internal curing peak and the evolution of the T_2_ spectrum—indicate that soda residue facilitates a dynamic water absorption desorption process. This behavior effectively alleviates internal drying, promotes further cement hydration, collectively improving the mechanical and durability properties. This work not only validates the high-value utilization of soda residue but also provides a mechanistic understanding of its dual role in concrete.

## 1. Introduction

With the rapid development of infrastructure worldwide, cement-based materials, as the most essential construction materials, are facing increasingly high demands for workability, durability, and overall performance [1]. Conventional external curing methods are often ineffective in replenishing water deep within the concrete matrix, while chemical admixtures or supplementary materials like fly ash only partially mitigate these issues [2,3]. A more promising strategy involves incorporating internal curing agents that act as built-in water reservoirs, supplying moisture to counteract capillary pressure and shrinkage [4].

Internal curing materials are broadly categorized into superabsorbent polymers (SAPs) [5] and porous inorganic solids [6]. SAPs absorb water via hydrophilic functional groups in a three-dimensional network [7], whereas porous inorganic materials retain water through microporous structures. Although both have been widely studied, along with other materials such as rice husk ash [8], diatomite [9] and red mud [10], they present certain limitations. Porous inorganic materials often exhibit low water absorption and may impair strength and modulus at higher dosages [11]. SAPs effectively mitigate early shrinkage but face challenges in dispersion control and may leave voids after water release, potentially compromising mechanical properties and durability [12,13]. These limitations highlight the need for alternative internal curing agents that balance efficiency with minimal adverse effects.

Concurrently, sustainable construction practices necessitate the utilization of industrial by-products. Soda residue, a bulk solid waste generated from the ammonia-soda process, poses considerable environmental challenges. Global production of soda ash amounted to approximately 63.37 million tons in 2022, with China accounting for 29.20 million tons [14]. Projected to reach 74.07 million tons by 2030, soda residue is primarily disposed of via landfilling, resulting in soil salinization and water contamination that adversely affect vegetation growth [15,16]. Moreover, soda residue contains high levels of soluble salts, particularly chlorides [17,18] and magnesium salts [19], which can contaminate groundwater and pose risks to human health and ecosystem [20]. Current recycling approaches, including backfilling, geopolymer synthesis, and soil amendment, utilize only 3–4% of the total soda residue output [19]. Therefore, the development of efficient and scalable soda residue recycling technologies is urgently needed.

Incorporating soda residue into construction materials presents a promising recycling pathway, with existing research primarily exploring its role as a supplementary cementitious material [21,22,23]. Studies indicate that within an optimal dosage range of 6–20%, soda residue can enhance early-age strength due to its alkaline and sulfate components promoting ettringite formation [13,24,25]. However, a critical dosage threshold exists, beyond which excessive soda residue content (>20% in paste or >10% in concrete) leads to strength reduction primarily attributable to cement dilution effects [12]. The influence of soda residue on durability is governed by more complex, often competing, mechanisms that are highly dosage-dependent. Regarding chloride ingress resistance, an appropriate soda residue dosage can promote pore refinement and chemical binding through the formation of additional C-(A)-S-H gels and Friedel’s salt [26,27,28,29], yet excessive addition may impair impermeability due to reduced C-S-H content and increased crystallization pressure [29,30]. Similarly, for sulfate attack resistance, the beneficial matrix densification from accelerated hydration can be counterbalanced by potential instability induced by expansive reactions from carbonate components and portlandite consumption during Friedel’s salt formation [24,31,32,33]. Frost resistance also demonstrates significant composition dependence [34], underscoring that optimizing the soda residue dosage and potentially combining it with complementary mineral admixtures are crucial strategies for synergistically enhancing the overall durability performance of soda residue-incorporated cementitious systems.

However, a significant knowledge gap persists regarding the fundamental mechanisms of soda as an internal curing agent. While pioneering studies have confirmed its microporous structure [35] and demonstrated its efficacy in reducing early-age autogenous shrinkage, even inducing early expansion despite a slight workability loss [13]. These investigations remain largely phenomenological. For instance, research shows chloride-washed soda residue can absorb 1.2 times its mass and release 80% of the water, effectively reducing shrinkage in UHPC [36], while a 7.5% cement replacement can achieve nearly 50% shrinkage reduction without strength loss [37]. Crucially, a comprehensive understanding of the underlying water kinetics (i.e., the dynamic absorption, storage, and release behavior) and its explicit correlation with long-term macroscopic performance (e.g., durability, frost resistance) over the full life cycle is still lacking. Most existing work focuses on early-age effects, leaving the moisture transport mechanisms and their long-term implications insufficiently elucidated.

To address these issues, this study hypothesizes that soda residue, through optimized processing via water-washing and fly ash-assisted grinding, functions as an efficient internal curing agent by modulating its water kinetics, which governs the enhancement of macroscopic concrete properties. Accordingly, the experimental program was designed with the following objectives. Firstly, to evaluate the effects of the soda residue-based internal curing material on the compressive strength, shrinkage behavior, and frost resistance of concrete. Subsequently, to characterize its water absorption desorption kinetics using low-field nuclear magnetic resonance (LF-NMR) and hydration heat analysis. Ultimately, to establish a direct correlation between these water transport mechanisms and the macro-performance, thereby clarifying the competition between the beneficial internal curing effects and potential weak interfaces. This work aims to provide a mechanistic foundation for the high-value utilization of soda residue and to advance the fundamental understanding of internal curing in sustainable cement-based materials.

## 2. Materials and Methods

### 2.1. Raw Materials and Mix Proportions

The cement used in this study was P·O 42.5 ordinary Portland cement (supplied by bbmg jidong cement group Co., Ltd., Tangshan, China). The fly ash was Class F ash (obtained from Tangshan Fengrun Jinshengyuan Fly Ash Storage Warehouse, Tangshan, China), with a 45 μm sieve residue of 28.3%, water demand ratio of 102%, and loss on ignition of 7.4%. The chemical compositions of fly ash and cement are summarized in Table 1. Fine aggregate consisted of Zone II medium sand (natural river sand) with a fineness modulus of 2.48; coarse aggregate was continuously graded crushed stone with a particle size range of 5–20 mm.

**Figure 1:**
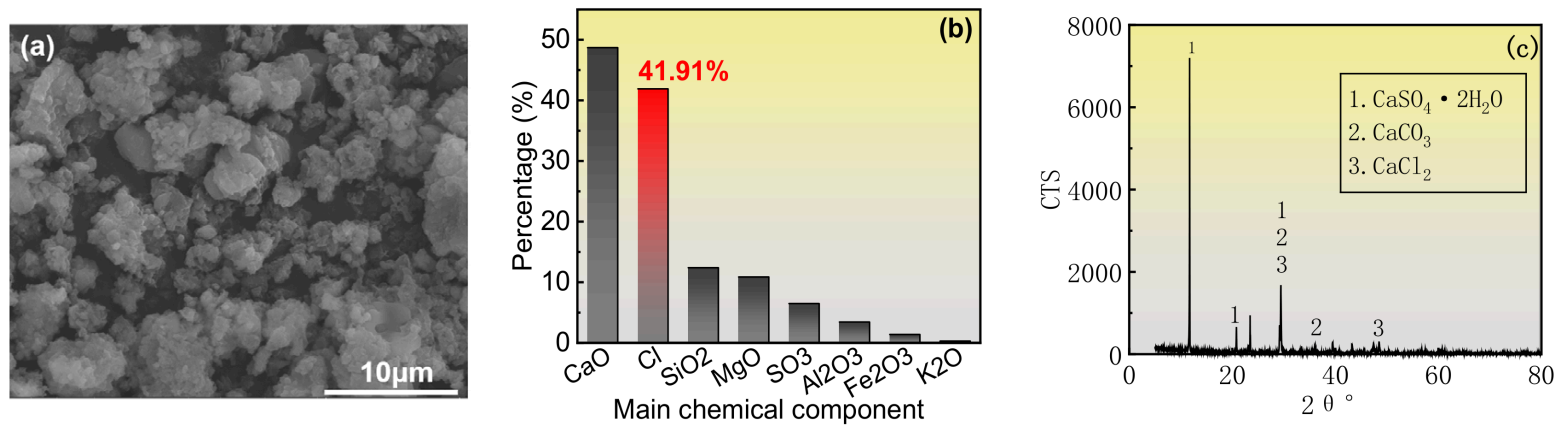
Basic properties of original soda residue: (**a**) microscopic morphology, (**b**) main chemical component and (**c**) XRD patterns.

**Figure 2:**
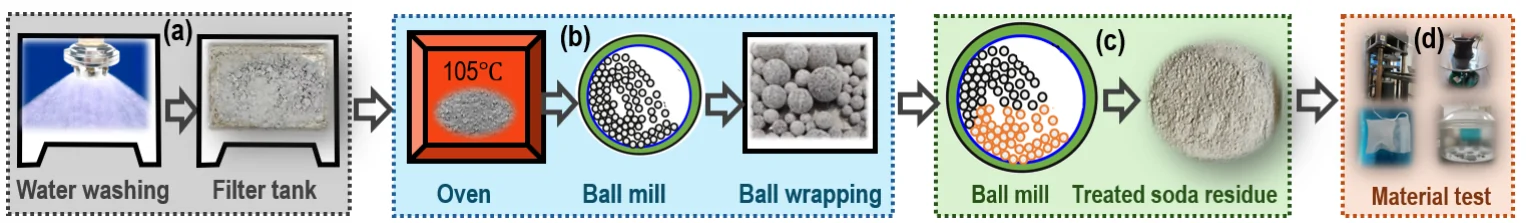
Process diagram for preparing soda residue: (**a**) washing to remove chloride, (**b**) drying and ball milling and (**c**) grinding with fly ash to obtain soda residue internal curing material. (**d**) testing the physical and chemical properties of internal curing materials.

**Figure 3:**
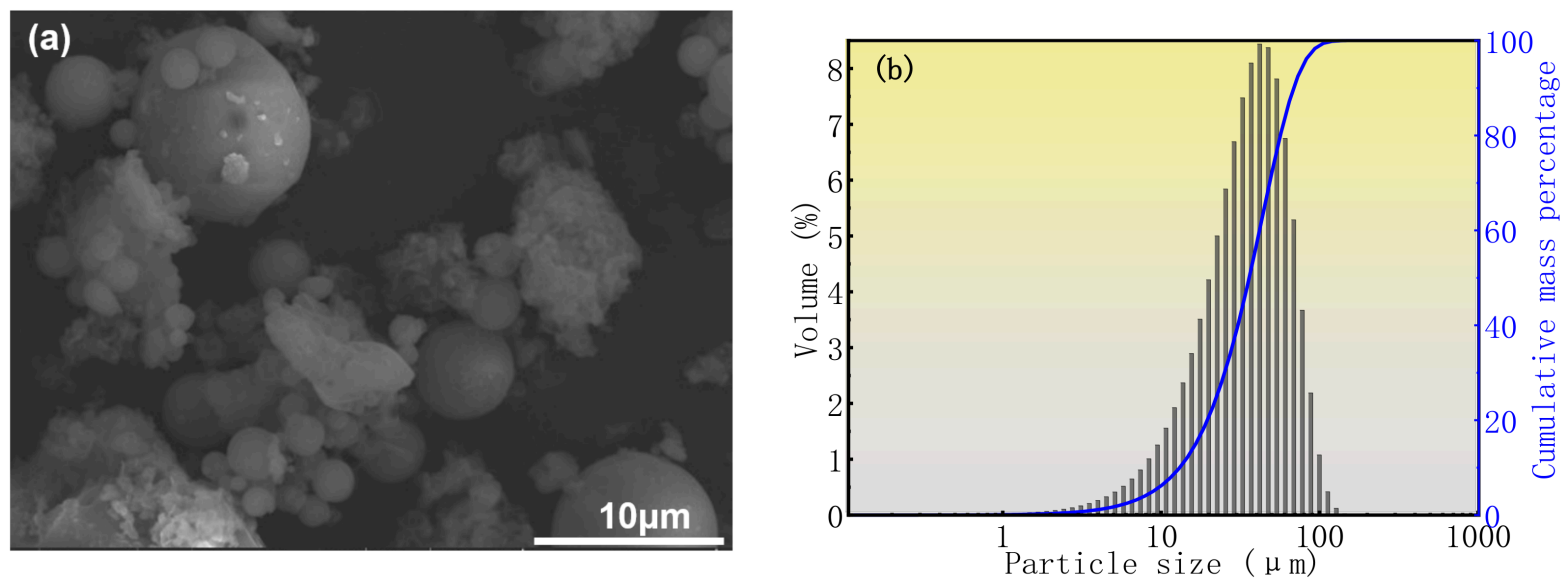

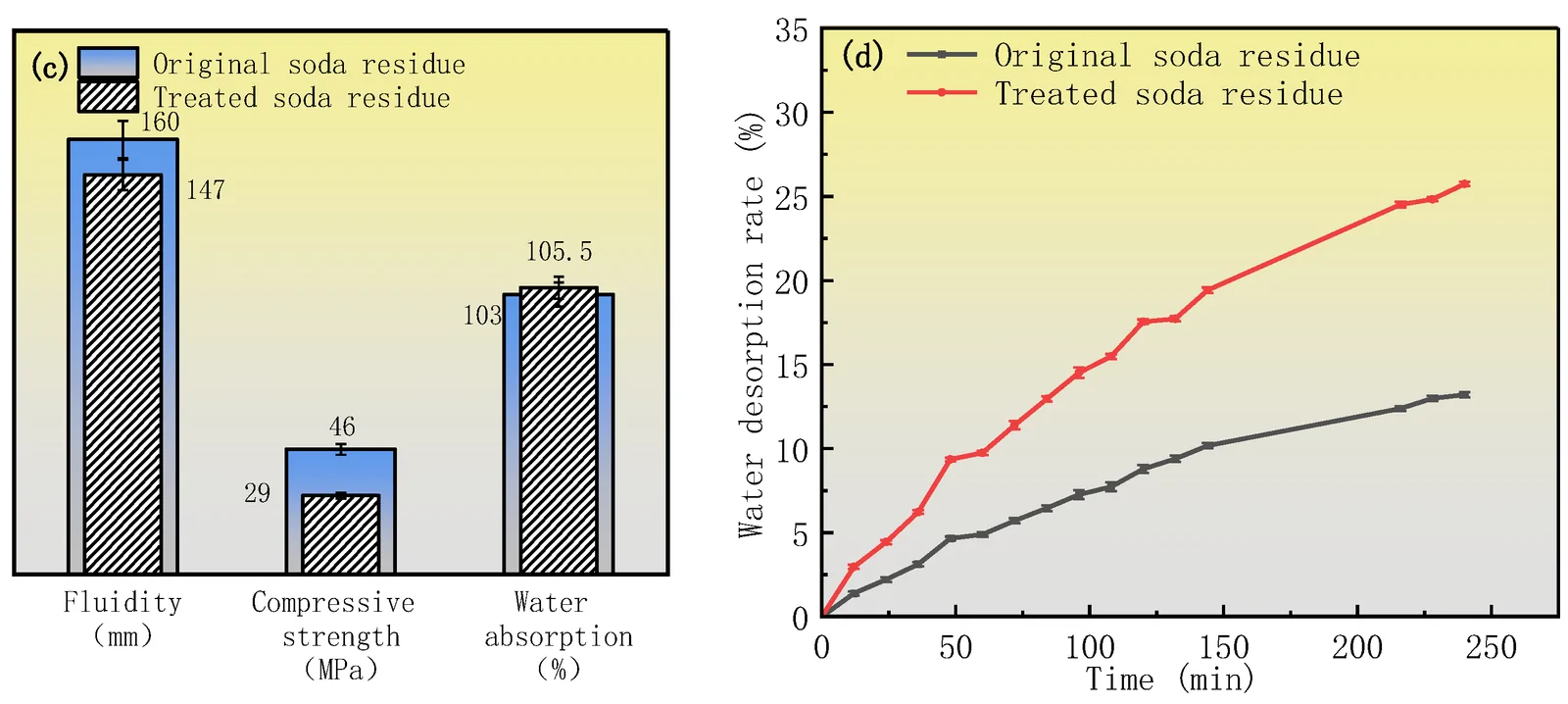
The material properties of treated soda residue used in this project: (**a**) microscopic morphology, (**b**) particle size distribution curve, (**c**) fluidity, compressive strength and water absorption properties, and (**d**) water desorption properties.

The original waste soda residue used in this study was obtained from Tangshan Sanyou Chemical Co., Ltd., Tangshan, China presenting as a light grayish-white wet block with a moisture content ranging from 32% to 52%. The basic properties of the raw material are presented in Figure 1. The original waste soda residue has a water absorption rate of 103%. The density of the original waste soda residue is 2.37 g/cm^3^. To reduce the chloride ion content, which exceeded 40%, a water-washing treatment was applied: the original soda residue was mixed with laboratory water at water-to-solid mass ratios of 8:1 and 10:1, stirred, and filtered. The filter residue was then dried at 105 °C to constant mass, yielding two types of treated soda residue. Based on a comprehensive evaluation of water absorption release behavior, strength activity, and flowability, the material washed at a water-to-solid ratio of 8:1 was selected as the precursor. After drying, it was ball-milled with fly ash at a mass ratio of 2:1 for 2 min to produce the final soda residue-based internal curing material (hereafter referred to as soda residue). The preparation process and related performance tests are illustrated in Figure 2, and the material properties of the treated soda residue are summarized in Figure 3. Although the treated material exhibits reduced strength and activity compared to the original soda residue, its chloride ion content is significantly lowered, and it demonstrates favorable water absorption and release characteristics. Accordingly, the treated soda residue (i.e., the soda residue internal curing material described herein) was incorporated into both concrete and slurry samples in the subsequent experiments.

**Figure 4:**
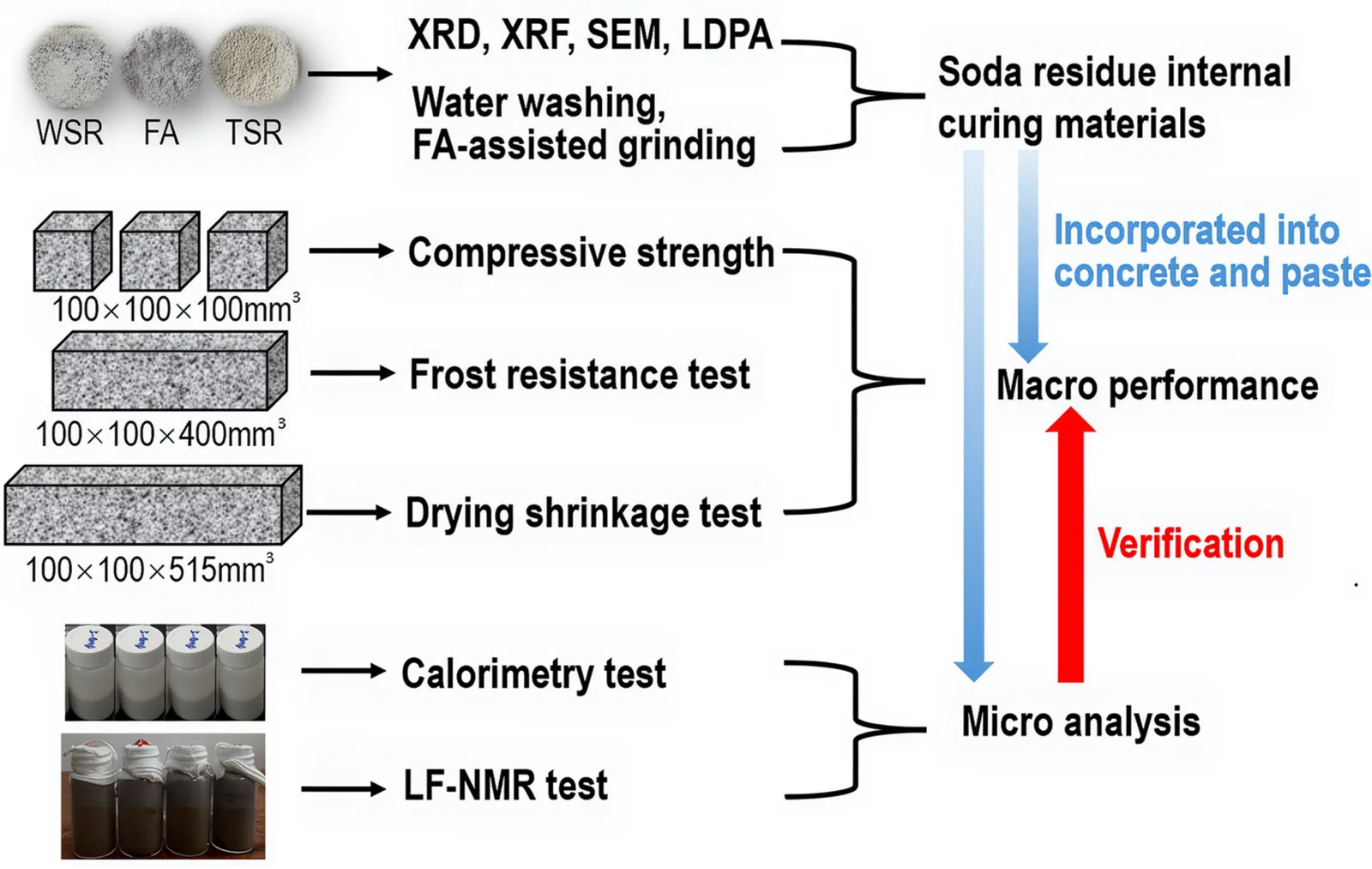
The overall experimental procedure.

Based on the results of previous orthogonal experiments and range analysis, the optimal dosage of mineral admixture in ordinary concrete with a water to cement ratio of 0.5 was determined to be 30%. On this basis, the soda residue content was set at 0%, 5%, 10%, and 15%, with the fly ash content adjusted accordingly to maintain a total mineral admixture content of 30%. Treated soda residue replaces part of the fly ash in the system by equal mass. The corresponding mix proportions were designated as PC0, PC05, PC10, and PC15, respectively. The detailed mix proportions are provided in Table 2. Three parallel specimens were used for each test of macroscopic mechanical properties, shrinkage and frost resistance. The overall experimental procedure is illustrated in Figure 4 and more details are given in the following.

### 2.2. Drying Shrinkage Test

The drying shrinkage of concrete was evaluated following GB/T 50082-2024 [38]. Prismatic specimens with dimensions of 100 mm × 100 mm × 515 mm were prepared and subjected to standard curing for 3 days prior to demolding. After demolding, the specimens were transferred to a controlled environment maintained at 20 ± 2 °C and 60 ± 5% relative humidity. Length changes at various ages were measured using a horizontal concrete shrinkage and expansion apparatus (HSP-540, with a precision of 0.01 mm, Beijing Zhongjiao Construction Instrument Technology Development Co., Ltd., Beijing, China), and the drying shrinkage value was calculated according to the standard formula.

### 2.3. Frost Resistance Test

The resistance of concrete was evaluated using the rapid freezing method in accordance with GB/T 50082-2024. Prismatic specimens measuring 100 mm × 100 mm × 400 mm were prepared. After 24 days of standard curing, the specimens were water-saturated for 4 days and subsequently placed in a TDR-16 concrete rapid testing chamber Cangzhou Xinmingsheng Testing Instruments Co., Ltd., Cangzhou, China. The center temperature of the specimens was controlled to cycle between −16 °C and 3 °C. After every 25 cycles, the mass and fundamental transverse frequency of the specimens were measured, and the mass loss rate and relative dynamic elastic modulus were calculated.

### 2.4. Calorimetry Test

The calorimetry test was performed using an eight-channel TAMAir microcalorimeter, TA Instruments, New Castle, DE, USA. Prior to testing, the raw materials were accurately weighed based on the binder and their respective heat capacities, and then placed into dedicated ampoule bottles as the test sample and reference sample, respectively. The samples were preconditioned at a constant temperature in the laboratory for 24 h. Subsequently, the corresponding amount of water was added according to the mix proportion and mixed uniformly. The ampoule bottles containing the paste and reference sample were then placed into the corresponding instrument channels, and the heat evolution was monitored continuously for 7 days.

### 2.5. LF-NMR Test

The early hydration process of cement paste incorporated with varying contents of soda residue was monitored in situ using a low-field nuclear magnetic resonance (LF-NMR) analyzer (PQ001, Suzhou Niumag Analytical Instrument Corporation, Suzhou, China). The sample preparation procedure was conducted as follows: raw materials were first precisely weighed according to the designated mix proportion. Water was then added, and the mixture was stirred manually at a high speed. Finally, the resulting paste was transferred into a 10 mL chromatographic bottle. The prepared sample in the chromatographic bottle was placed into the instrument. After signal stabilization, data were automatically collected at preset time intervals. The T_2_ relaxation spectrum was obtained via an inversion algorithm. The LF-NMR test parameters were set as follows: echo time = 0.06 ms, number of echoes = 12,000, waiting time = 3000 for PC0 and 3500 for PC10, and number of scans = 24. Throughout the testing process, the signal-to-noise ratio was maintained above 200, with number of scans adjusted as necessary to ensure data quality.

## 3. Results and Discussion

### 3.1. Shrinkage Properties

**Figure 5:**
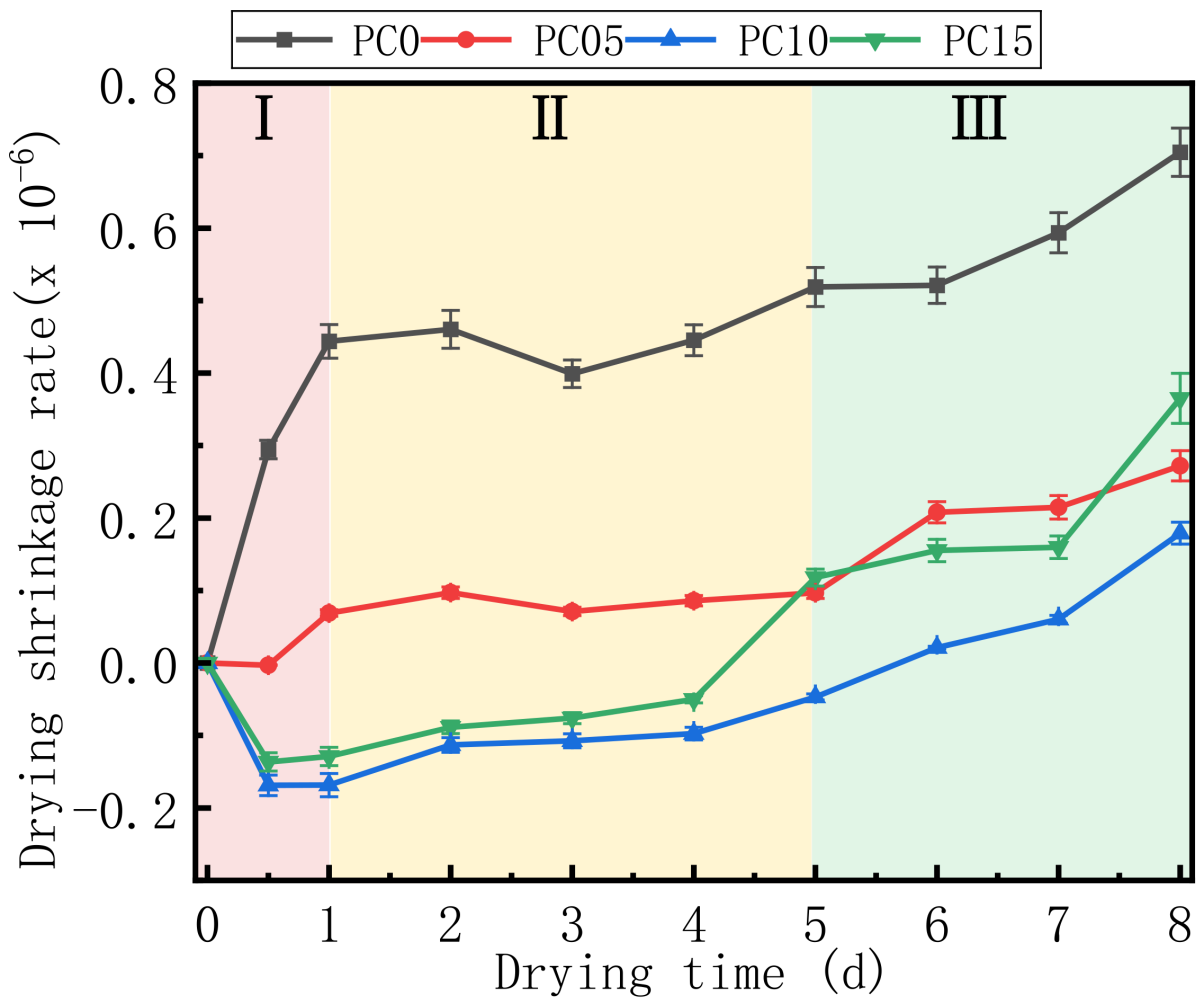
The influence of soda residue on the drying shrinkage performance of concrete.

Figure 5 illustrates the influence of soda residue content on the drying shrinkage behavior of concrete. The drying shrinkage of the control group PC0 increases continuously over time and can be divided into three distinct stages: during the initial stage (0–1 day), rapid evaporation of free water leads to a sharp increase in shrinkage; in the mid-term (1–5 days), the growth rate slows but continues to accumulate; in the later stage (>5 days), shrinkage rises again due to further internal humidity reduction and structural contraction of hydration products, eventually reaching the maximum value. This pattern indicates that conventional concrete is highly sensitive to dry environments.

The incorporation of soda residue significantly alters the early-age shrinkage behavior of concrete. They all exhibited reduced shrinkage at early ages, with PC10 and PC15 even showing slight expansion, and PC10 demonstrating the most pronounced negative shrinkage amplitude. This phenomenon can be primarily attributed to the dual effects of soda residue: first, its porous structure absorbs and stores water, which is gradually released under dry conditions, thereby delaying the decline in internal humidity and mitigating capillary negative pressure (internal curing effect) [13]; second, sulfate ions present in soda residue promote the formation of expansive ettringite, effectively compensating for shrinkage.

With increasing age, the shrinkage of specimens containing soda residue shifts from negative to positive. Nonetheless, by day 7, their shrinkage strains remain significantly lower than that of PC0, with reductions of 65.4%, 80.3%, and 38.4%, respectively, confirming the sustained internal curing effect of soda residue. However, excessive soda residue content (PC15) accelerates shrinkage in later stages. At day 8, although its shrinkage remains below that of PC0, it is notably higher than PC05, indicating that the mitigation effect does not increase monotonically with dosage, but arises from the combined action of internal curing and the “clinker dilution effect”. An appropriate dosage (10%) effectively suppresses shrinkage through sustained moisture release, while excessive addition increases water demand and porosity, creating additional moisture escape paths and thereby compromising shrinkage control.

### 3.2. Resistance

**Figure 6:**
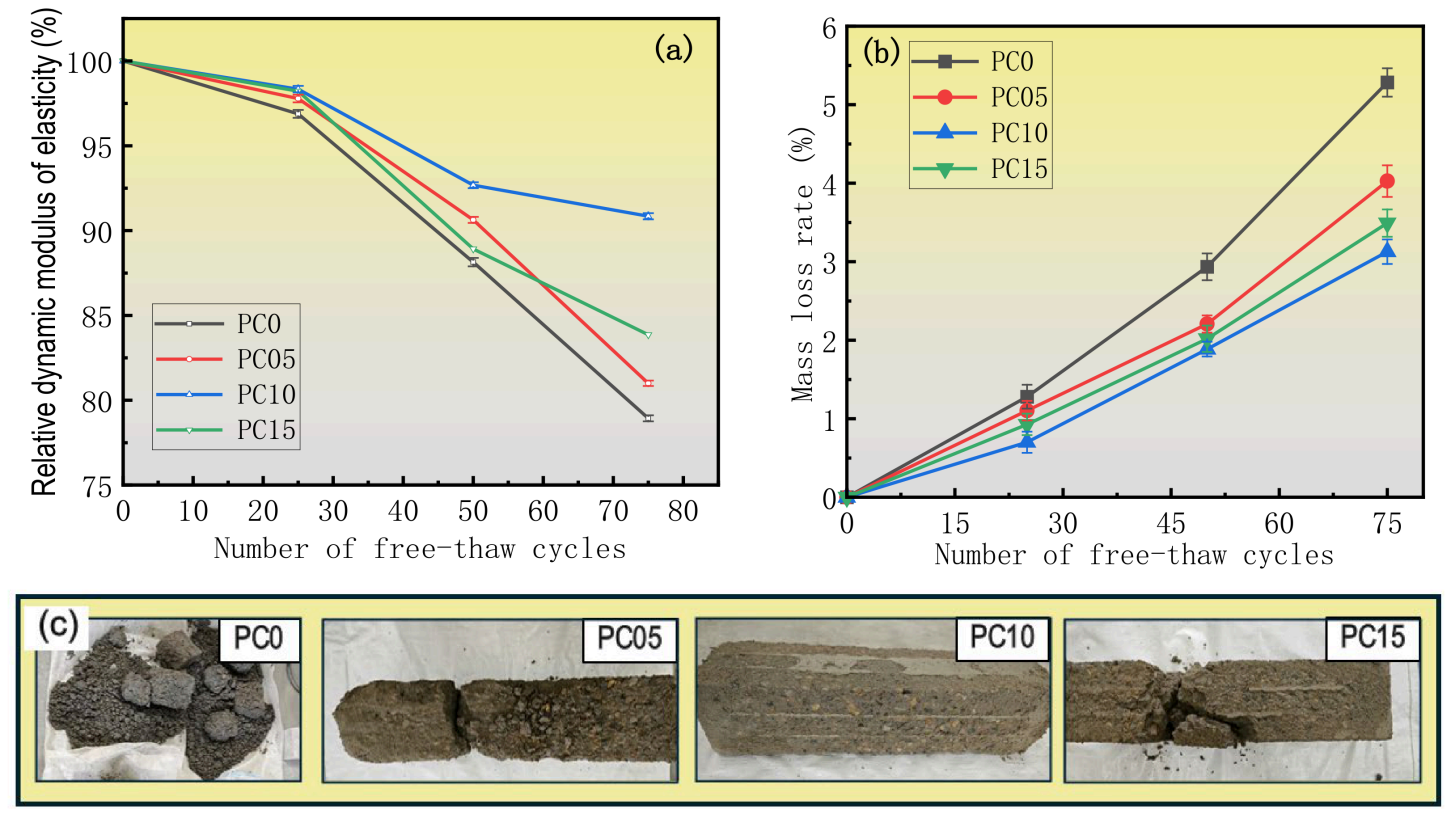
The effect of soda residue on the resistance of concrete: (**a**) mass loss rate, (**b**) relative dynamic modulus of elasticity and (**c**) surface scaling after 50 cycles.

Figure 6 illustrates the variation in relative dynamic elastic modulus, mass loss rate and surface scaling of concrete under cycling. With increasing number of cycles, the dynamic elastic modulus of all mixtures decreased continuously, but the extent of attenuation differed markedly. The reference mixture PC0 exhibited the most rapid deterioration (Figure 6c); after 50 cycles, its relative dynamic elastic modulus dropped below 80%, indicating severe internal damage. The incorporation of soda residue significantly delayed the reduction in relative dynamic elastic modulus. Among all mixtures, PC10 maintained the highest modulus and demonstrated the best frost resistance throughout the process.

The variation in mass loss rate aligns closely with the trend observed in the relative dynamic elastic modulus. After 75 cycles, the mass loss rate of the reference mixture PC0 exceeded 5%, accompanied by severe surface mortar spalling. In contrast, the mass loss of specimens incorporating soda residue was significantly lower. Specifically, PC10 exhibited a 41.5% reduction in mass loss compared to PC0, indicating superior surface structural integrity and enhanced resistance to induced spalling damage.

The improvement in frost resistance of soda residue-modified concrete is attributed to the combined action of multiple mechanisms, among which the continuous internal curing effect serves as one of the main contributing factors. Water stored within the soda residue particles is gradually released during the later stages of hardening, promoting further hydration of unhydrated cementitious materials. This process refines the pore structure and reduces the volume of harmful pores, thereby effectively mitigating the expansion pressure generated by pore water freezing during cycles and suppressing the initiation and propagation of microcracks. In addition, residual soluble salt components in soda residue, such as chlorides and sulfates, may also exert a dual influence on the freezing behavior of the system. On one hand, a small amount of soluble salts can lower the freezing point of the pore solution, alleviating frost heave pressure to a certain extent. On the other hand, excessive salts may induce crystallization pressure in pores or promote the formation of harmful expansive phases, which could potentially deteriorate frost resistance. From a dosage perspective, PC10 exhibited optimal performance in terms of modulus retention and mass loss control, indicating that at this dosage, the positive effects of internal curing and matrix densification achieved the best synergy. When the dosage increased to 15%, the excessive soda residue may introduce more initial defects due to the clinker dilution effect, partially counteracting the benefits of internal curing. As a result, although PC15 still showed improved frost resistance compared to the reference PC0, its performance was inferior to that of PC10. It should be clarified that the durability assessment in this work is limited to the resistance of concrete. Other critical durability metrics of the soda residue-modified system, including chloride penetration resistance, sulfate attack resistance, carbonation resistance, steel corrosion risk and long-term leaching behavior, have not been systematically investigated. The above multi-dimensional durability evaluation will be conducted in our follow-up studies.

### 3.3. Hydration Heat

**Figure 7:**
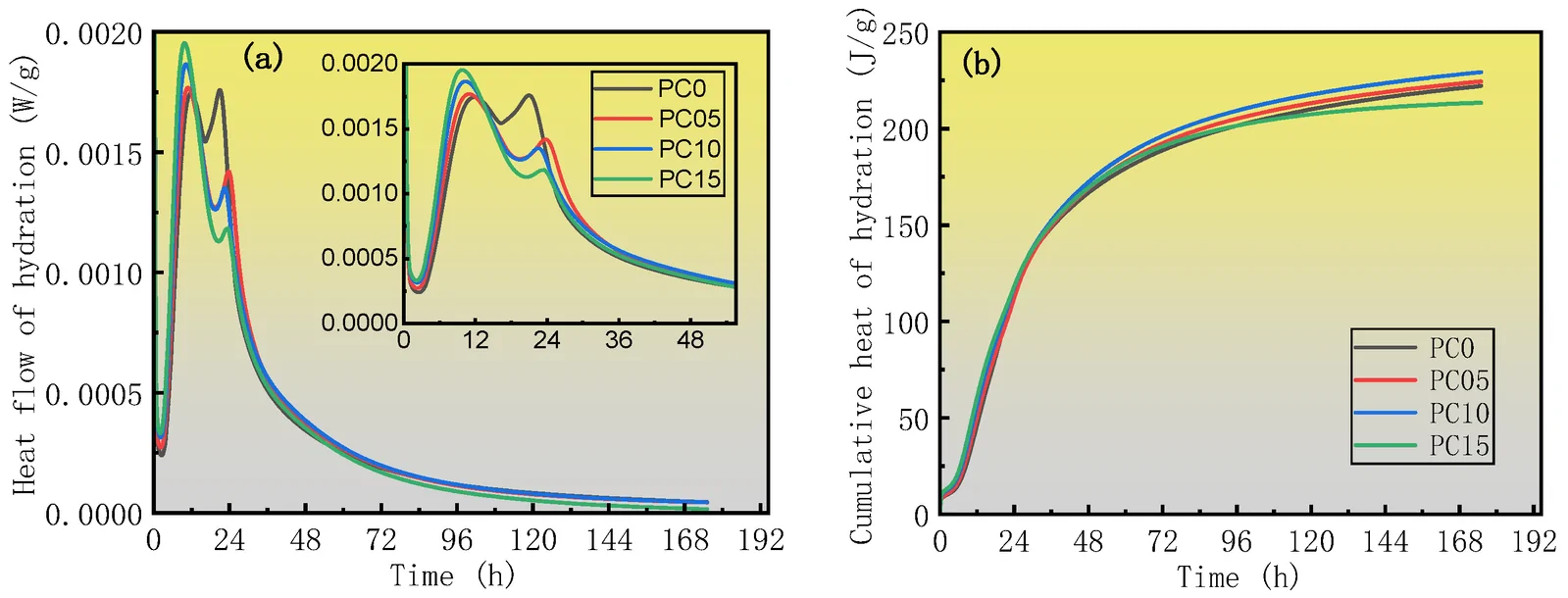
(**a**) The hydration heat release rate curve of different soda residue dosages in paste samples. (**b**) The accumulated heat release curve of different soda residue dosages in paste samples.

Figure 7 presents the hydration heat release characteristics of concrete with varying soda residue contents. The heat release rate curves of all mixtures (Figure 7a) display typical multimodal features, indicating that soda residue does not fundamentally alter the hydration mechanism but modulates its progression. Specifically, with increasing soda residue content, the occurrence of the main hydration peak shifts slightly earlier, whereas the third peak which is associated with the pozzolanic reaction of mineral admixtures at later ages is delayed. This delay is attributed to the partial replacement of fly ash by soda residue, which has lower chemical activity. As a result, the amount of reactive components available for the pozzolanic reaction in the later stages is reduced, slowing the reaction kinetics during this period [39,40,41].

The accumulated heat release curves in Figure 7b show limited differences among the mixtures during the early stage (<24 h), but significant divergence at later ages. The cumulative heat release of PC10 exceeded that of PC0, whereas PC15 exhibited a notable reduction. This trend reveals the dual role of soda residue: an appropriate dosage (10%) enhances the alkalinity of the pore solution through its alkaline activation effect and continuously supplies moisture via internal curing [2], collectively promoting the pozzolanic reaction of fly ash and the ongoing hydration of cement. In contrast, an excessive dosage (15%) leads to the replacement of a substantial amount of reactive fly ash. Since soda residue itself contributes little to heat release, its positive effects cannot compensate for the heat reduction resulting from the diminished fly ash reaction.

In summary, the influence of soda residue on the hydration heat release of concrete stems from the interplay between its promoting effect and dilution effect. At an appropriate dosage (10%), the beneficial contributions of alkali activation and internal curing prevail, moderately enhancing the total heat release. However, when the dosage becomes excessive (15%), the dilution effect—resulting from the replacement of a significant portion of reactive fly ash—dominates, leading to a reduction in the overall degree of hydration and cumulative heat release. Therefore, a soda residue content of 10% represents the optimal dosage, maximizing the beneficial contributions while maintaining a favorable balance in the hydration process.

### 3.4. Water Kinetics

#### 3.4.1. Water States Distribution

**Figure 8:**
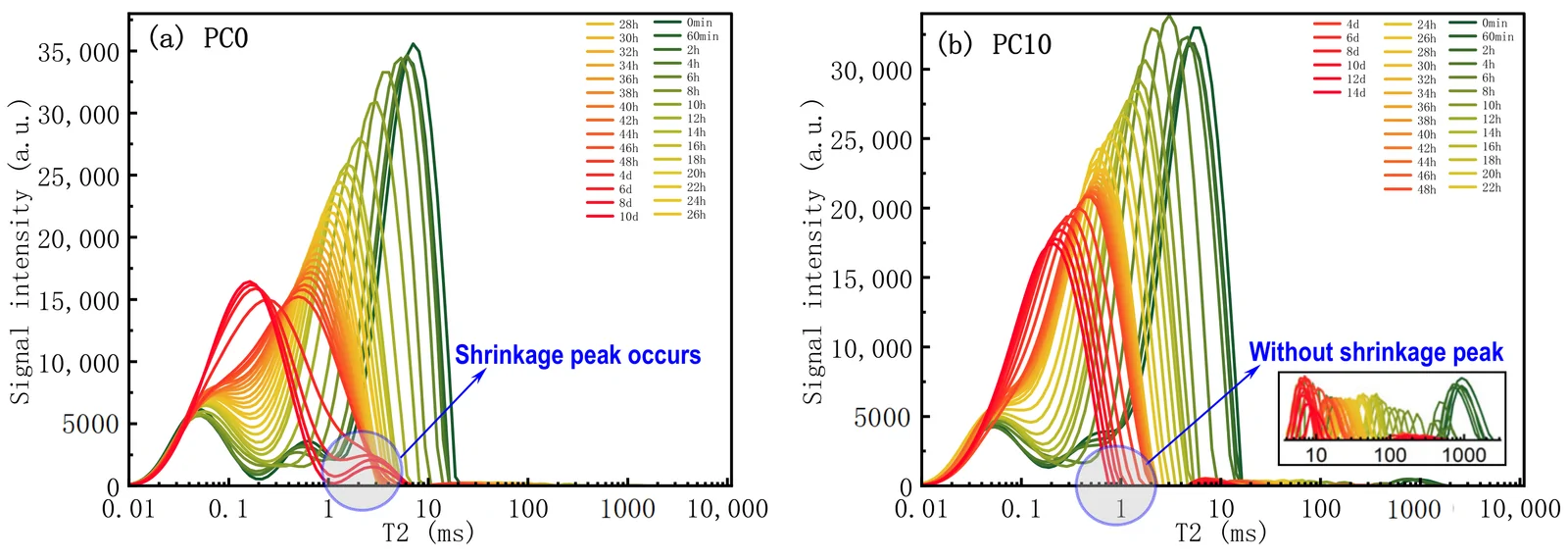
(**a**) T_2_ spectra evolution for PC0. (**b**) T_2_ spectra evolution for PC10.

To elucidate the micro-level mechanism by which soda residue internal curing (at a dosage of 10%) enhances the macroscopic properties of concrete, LF-NMR was employed to compare the T_2_ relaxation spectra of PC0 and PC10, as shown in Figure 8. The T_2_ spectrum peak position reflects the state of water molecules: shorter T_2_ values correspond to bound water in smaller pores, while longer T_2_ values indicate free water in larger pores. The peak area quantifies their relative content [42]. Consequently, the evolution of the T_2_ spectrum provides an intuitive representation of internal water migration and pore structure refinement.

The T_2_ spectra of both mixtures exhibit multi-peak characteristics. As hydration proceeds, the main peaks shift toward shorter T_2_ values, reflecting the general trend of pore refinement and structural densification due to the accumulation of hydration products. However, key differences are observed between the two systems: first, PC0 shows a distinct “shrinkage peak” at day 5, which is absent in PC10, indicating that soda residue mitigates shrinkage stress through sustained internal water release. Second, the long-T_2_ signal intensity in PC10 remains significantly higher than that in PC0, further supporting the role of internal curing in maintaining moisture availability within the pore structure. Together, these findings confirm the internal curing function of soda residue: its porous structure absorbs and stores water during early stages and gradually releases it later. This mechanism delays the drop in internal relative humidity, reducing the driving force for drying shrinkage, while also supplying moisture for continued hydration of unreacted cementitious particles. As a result, it promotes additional C-S-H gel formation, further refines the pore structure, and suppresses undesirable shrinkage peaks. These observations elucidate the microstructural basis for the superior volume stability and frost resistance of the PC10 mixture. Quantitative analysis of the water absorption and release behavior of soda residue is provided in Section 3.4.4.

#### 3.4.2. Temporal Variations in T_2_ Spectral Features

**Figure 9:**
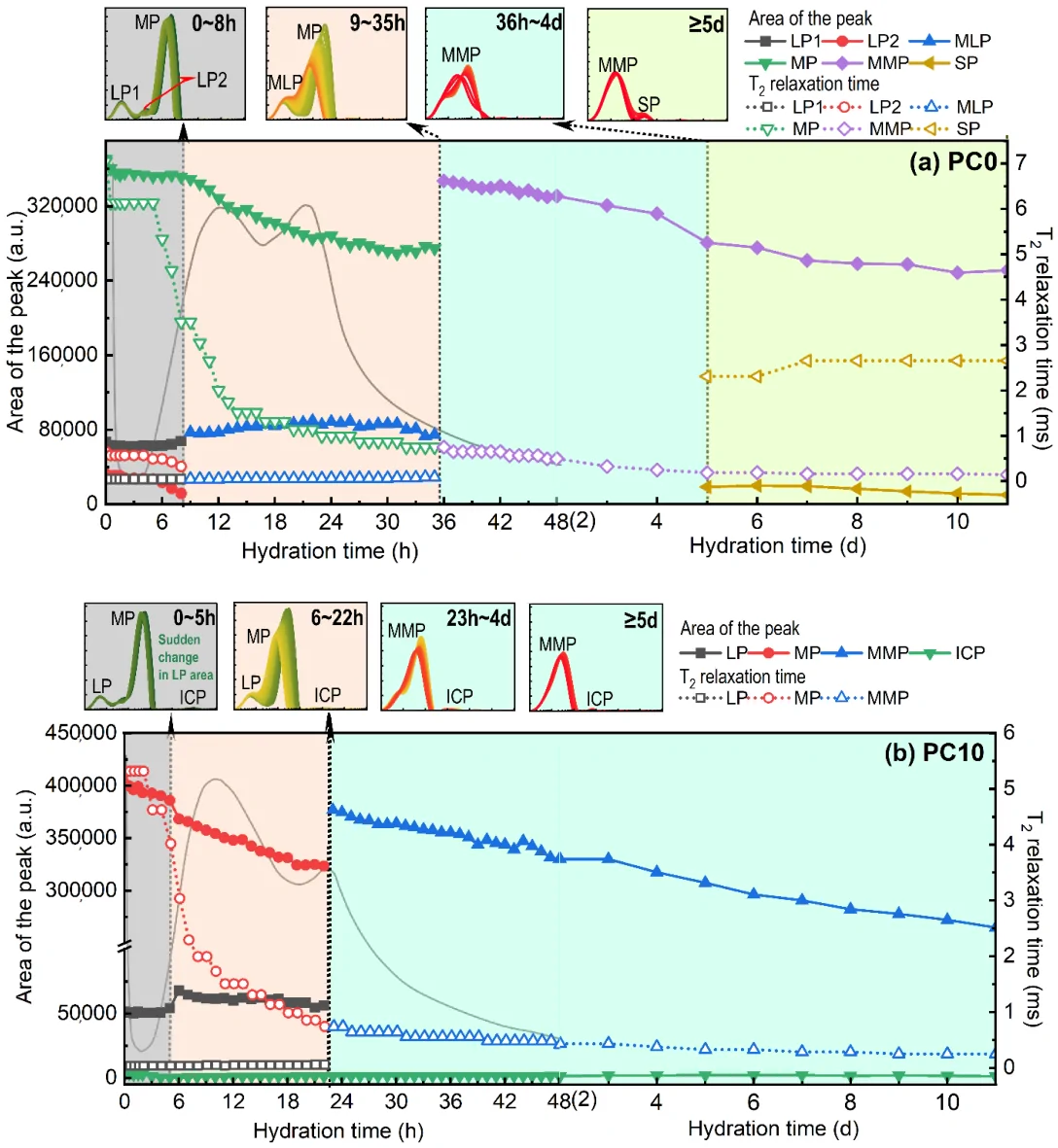
(**a**) Evolution of peak area and T_2_ peak values during the hydration process of PC0. (**b**) Evolution of peak area and T2 peak values during the hydration process of PC10.

From an observational perspective, the initial T_2_ spectrum of PC0 displays four characteristic peaks, designated as LP1 (left peak 1), LP2 (left peak 2), MP (main peak), and FP (free water peak). With ongoing hydration, LP1 and LP2 gradually merge to form MLP (merged left peak), which subsequently combines with MP to yield MMP (merged main peak). In later stages, a new peak, SP (shrinkage peak), emerges on the right side of MMP. In contrast, the initial T_2_ spectrum of PC10 exhibits only three peaks, labeled as LP, MP, and ICP (internal curing water peak induced by soda residue). Throughout hydration, LP and MP progressively merge into MMP, with both the peak area and corresponding T_2_ value decreasing continuously. Notably, the incorporation of soda residue leads to the appearance of the ICP peak in PC10, while no SP peak is observed. The fusion and separation of peaks in the T_2_ spectrum reflect underlying changes in internal chemical reactions. By extracting the peak areas and corresponding T_2_ values from Figure 8a and integrating them with the hydration heat release rate curve in Figure 7a, a multidimensional evolution diagram of the hydration process was constructed, as presented in Figure 9.

Based on the evolution of the T_2_ spectrum in Figure 9a, the following conclusions can be drawn: (1) the variation in the T_2_ value of the main peak corresponds well with the hydration exothermic process—its stable phase aligns with the induction period, the rapid decline coincides with the main hydration peak stage, and the gradual decrease matches the slow reaction period; (2) the merging of LP and MP during the main hydration stage suggests a blurring distinction between gel water and capillary water, reflecting rapid growth and bridging of C-S-H gel, likely corresponding to the formation of a continuous network by needle-like C-S-H products; (3) the gradual attenuation of the MMP signal indicates ongoing densification of the gel structure, consistent with microstructural evolution in the slow hydration stage; (4) the emergence of a small SP peak in PC10 at 4 days, which disappears after soda residue addition, implies its association with shrinkage behavior.

As illustrated in Figure 9b, the T_2_ value of the main peak in PC10 also maintains a strong correlation with the hydration heat release process even after the incorporation of soda residue. Notably, no shrinkage peak was observed throughout the 11-day hydration period, which is likely attributable to the internal curing effect provided by the soda residue. This effect mitigates self-desiccation and associated shrinkage stress through continuous water release. It is also noteworthy that the merging of the LP and MP peaks into MMP coincides with the secondary exothermic peak in the hydration heat curve, suggesting a close relationship with the pozzolanic reaction. The pozzolanic reaction accelerates the consumption of reactive components and the formation of gel products, facilitating the integration of originally distinct capillary and gel water systems into a denser microstructure, thereby promoting the early formation of MMP.

#### 3.4.3. Relationship Between T_2_ and Compressive Strength

**Figure 10:**
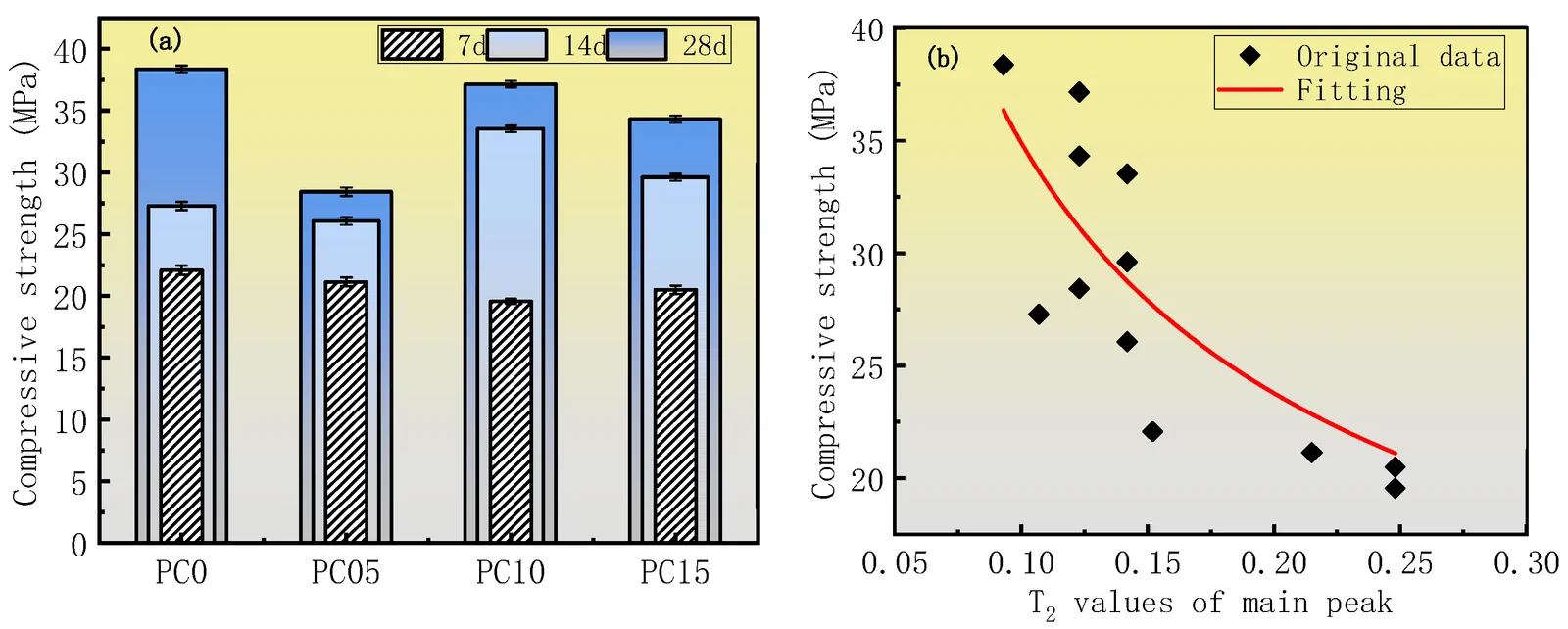
(**a**) Compressive strength of different test blocks at different ages. (**b**) Correlation between compressive strength and T_2_ values of main peak.

Figure 10a demonstrates the influence of soda residue content on the compressive strength of concrete. At 7 days, the compressive strength decreases gradually with increasing soda residue content. However, as the curing age extends to 14 and 28 days, the compressive strength first increases and then decreases, peaking at a soda residue content of 10%. This behavior reflects a dynamic competition between the internal curing effect provided by soda residue and the weakening effect introduced by interfacial defects. At the 10% dosage, the internal curing effect dominates, mitigating self-desiccation through sustained water release and promoting later-stage hydration, thereby enhancing compressive strength. The strength gains at 14 and 28 days further confirm that soda residue continues to function as an internal curing agent beyond 7 days, which is consistent with the sustained water release behavior after 7 days reported in Section 3.4.4. Overall, soda residue improves the mechanical properties of concrete by optimizing internal moisture distribution and promoting microstructural densification during prolonged curing.

The peak T_2_ value serves as an indicator of the dominant pore size within the paste phase and is theoretically correlated with compressive strength [42]. As shown in Figure 10b, which compares the peak T_2_ values with the compressive strength data from Figure 10a, a general decreasing trend in compressive strength is observed with increasing T_2_ values, suggesting that coarsening of the pore structure adversely affects mechanical performance. However, the correlation is not particularly strong. This may be attributed to the fact that the LF-NMR measurements were conducted on paste samples of the same mix proportion, whereas the macroscopic strength of concrete is influenced by additional multi-scale factors, such as aggregates and the interfacial transition zone [43]. Consequently, it is challenging to establish a direct functional relationship between the paste-scale T_2_ data and the overall concrete strength. Nevertheless, the observed negative correlation remains physically meaningful, indicating that refining the pore structure of the paste phase is an important mechanism for enhancing the macroscopic mechanical properties of concrete. This insight also supports further multi-scale coupling analysis in concrete material design.

#### 3.4.4. Absorption and Desorption Properties of Soda Residue

**Figure 11:**
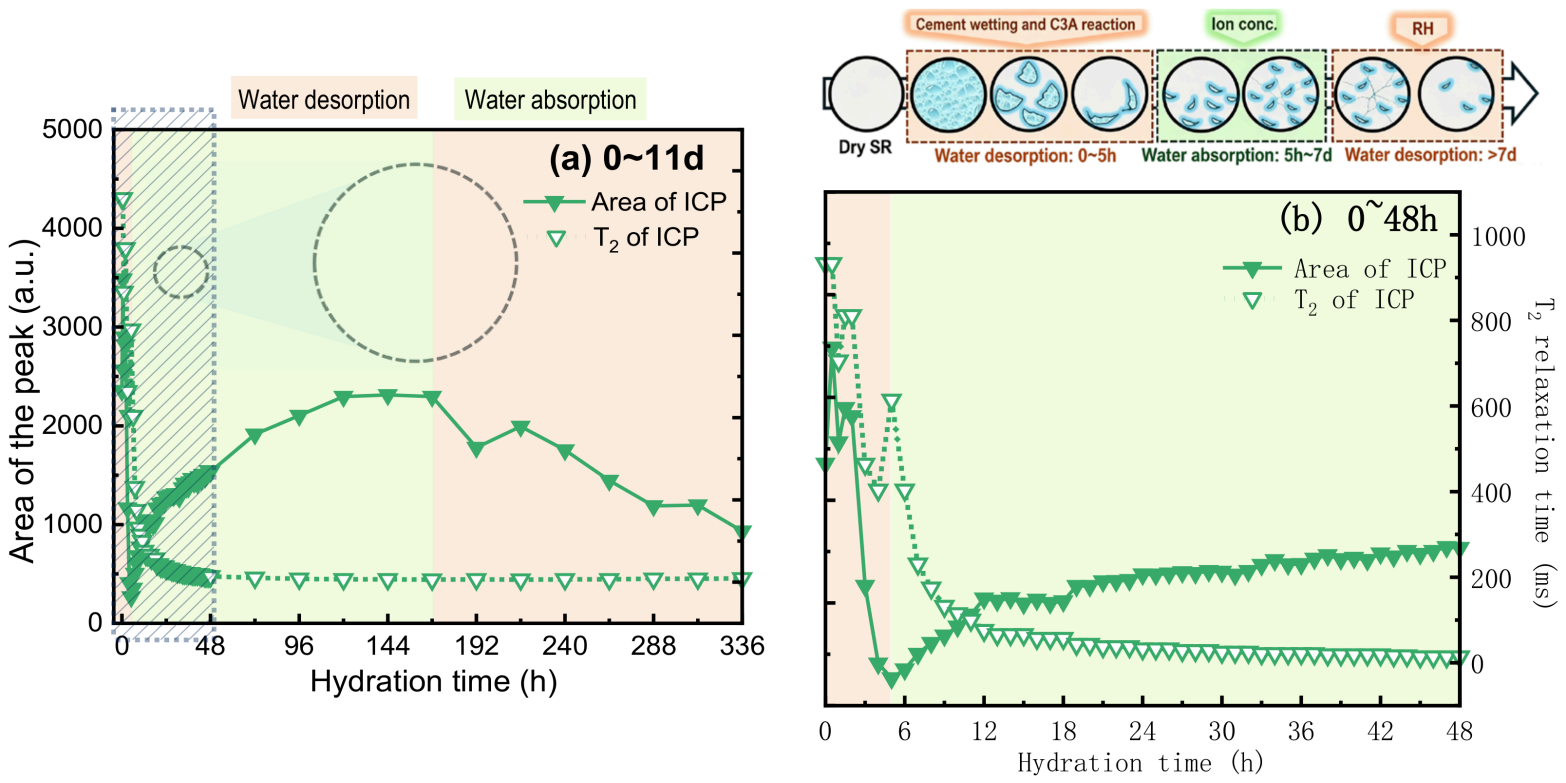
The kinetics of water absorption and desorption behavior of soda residue inside cement-based materials. (**a**) 0–11 d. (**b**) 0–48 h.

The aforementioned studies have shown that the improvement of macroscopic performance of concrete by soda residue is due to its dynamic water absorption and desorption behavior in cement-based materials. Taking PC10 as an example, analysis of its moisture transport over an 11-day hydration period (Figure 11) revealed that the water absorption and release process of soda residue occurs in two distinct stages. In the first stage, dry soda residue powder rapidly absorbs water upon mixing and gradually releases it within 1–5 h. The second stage involves slow water absorption from 5 h to 7 days, followed by sustained water release after 7 days. This well-timed and continuous release of water effectively mitigates internal drying shrinkage, promotes the formation of hydration products, and contributes to microstructural densification, leading to significant improvements in compressive strength and durability at the macroscopic level.

Regarding the driving mechanism behind the water absorption desorption behavior of soda residue, it is proposed that the release of water in the first stage (0–5 h) corresponds to the wetting of cement particles and the rapid reaction of aluminate minerals. During this period, cement particles in contact with the soda residue continuously absorb moisture from it, leading to a reduction in the water content of the residue. The subsequent water absorption behavior (after 5 h) is likely driven by a concentration gradient—after releasing water, the soda residue exhibits a higher internal concentration than the surrounding matrix, causing it to gradually absorb water from the cement paste. The water release observed after 7 days is primarily governed by a humidity gradient, as the matrix tends to dry out, prompting the soda residue to release moisture in a timely manner, thereby effectively fulfilling its role as an internal curing agent.

## 4. Conclusions

This study systematically evaluated the feasibility of utilizing waste soda residue as an internal curing agent in concrete and elucidated its comprehensive influence on macro performance and internal moisture kinetics. The principal conclusions are summarized as follows:

The addition of soda residue non-linearly influences the macroscopic properties of concrete. An optimal dosage of 10% soda residue was identified, resulting in the most favorable overall performance—characterized by minimal shrinkage, superior frost resistance, and the highest later-age compressive strength.

The performance of soda residue-modified concrete is governed by two competing mechanisms: the beneficial internal curing effect versus the detrimental effect of introduced weak interfaces. At the 10% dosage, the internal curing effect dominates. The sustained water release from soda residue mitigates self-desiccation, promotes prolonged hydration, and refines the microstructure, thereby overcoming the potential limitations associated with interfacial defects.

The evolution of the T_2_ spectrum (including the appearance, merging, and disappearance of peaks) is closely associated with the chemical processes of hydration. In early stages, the variation in the main peak T_2_ value corresponds well with the hydration heat release profile. In later stages, a distinct negative correlation exists between the main peak T_2_ value and compressive strength, underscoring the importance of pore structure refinement on mechanical performance. Notably, the soda residue-modified system displayed a clear internal curing peak, while the shrinkage peak evident in the control mixture was absent.

It is proposed that the efficacy of soda residue originates from its dynamic water absorption and release behavior within the cementitious matrix. Over 11 days of hydration, two distinct stages were identified: immediate water uptake upon mixing followed by release within 1–5 h, and a subsequent slow absorption phase from 5 h to 7 days, culminating in gradual release after 7 days. This well-timed water release alleviates internal drying shrinkage, promotes the formation of hydration products, and contributes to microstructural densification, which macroscopically manifests as enhanced compressive strength and durability.

Future research should first complete the systematic physical and chemical characterization of soda residue-based internal curing materials, including the quantitative chloride content before and after water washing, porosity, pore size distribution, and bulk density via mercury intrusion porosimetry (MIP) and other standard testing methods, to establish a comprehensive basic property database and facilitate cross-comparison among different internal curing agents. Future research should prioritize long-term performance assessments under real-world environmental conditions to evaluate the durability of soda residue-modified concrete over extended service periods. Further exploration is needed to determine its effectiveness in advanced cementitious systems such as ultra-high-performance concrete, which feature extremely low water-to-binder ratios. Additionally, establishing standardized protocols for the pretreatment and quality control of soda residue is essential to ensure its reliability and consistency as an internal curing material in industrial-scale applications.

## Figures and Tables

**Table 1:** Chemical composition of materials used in this project.

Materials	Na_2_O wt%	MgO wt%	Al_2_O_3_ wt%	SiO_2_ wt%	P_2_O_5_ wt%	SO_3_ wt%	Cl wt%	K_2_O wt%	CaO wt%	TiO_2_ wt%	MnO wt%	Fe_2_O_3_ wt%	Loss on Ignition %	Density g/cm^3^
Fly ash	0.20	0.92	36.12	48.80	0.36	0.73	0.01	1.51	3.83	1.70	0.06	5.14	7.4	2.21
Cement	0.25	3.99	7.42	21.14	0.07	4.73	0.07	1.33	57.56	0.69	0.13	2.51	4.6	3
Original soda residue	/	10.87	3.43	12.4	/	6.49	41.91	0.3	48.7	/	/	1.39	7.8	2.37
Treated soda residue	0.14	4.25	25.25	36.78	0.22	2.62	12.01	1.06	18.68	1.14	0.04	3.86	8.9	2.23

**Table 2:** Mix ratios of concrete used in this project.

	Cementitious Materials/%			Binder Content per m^3^/kg	w/b	Fine Aggregate per m^3^/kg	Coarse Aggregate per m^3^/kg
	Cement	FA	SR				
PC0	70	30	0	392	0.5	591	1257
PC05	70	25	5	392	0.5	591	1257
PC10	70	20	10	392	0.5	591	1257
PC15	70	15	15	392	0.5	591	1257

## Data Availability

The original contributions presented in this study are included in the article. Further inquiries can be directed to the corresponding author.
